# Characterization of a thermophilic cellulase from *Geobacillus* sp. HTA426, an efficient cellulase-producer on alkali pretreated of lignocellulosic biomass

**DOI:** 10.1371/journal.pone.0175004

**Published:** 2017-04-13

**Authors:** Laddawan Potprommanee, Xiao-Qin Wang, Ye-Ju Han, Didonc Nyobe, Yen-Ping Peng, Qing Huang, Jing-yong Liu, Yu-Ling Liao, Ken-Lin Chang

**Affiliations:** 1 School of Environmental Science and Engineering and Institute of Environmental Health and Pollution Control, Guangdong University of Technology, Guangzhou, China; 2 Department of Environmental Science and Engineering, Tunghai University, Taichung, Taiwan; 3 Key Laboratory of Urban Environmental and Health, Institute of Urban Environment, Chinese Academy of Sciences, Xiamen, China; 4 Institute of Environmental Engineering, National Sun Yat-Sen University, Kaohsiung, Taiwan; National Renewable Energy Laboratory, UNITED STATES

## Abstract

A themophilic cellulase-producing bacterium was isolated from a hot spring district and identified as *Geobacillus* sp. HTA426. The cellulase enzyme produced by the *Geobacillus* sp. HTA426 was purified through ammonium sulfate precipitation and ion exchange chromatography, with the recovery yield and fold purification of 10.14% and 5.12, respectively. The purified cellulase has a molecular weight of 40 kDa. The optimum temperature and pH for carboxymethyl cellulase (CMCase) activity of the purified cellulase were 60°C and pH 7.0, respectively. The enzyme was also stable over a wide temperature range of 50°C to 70°C after 5 h of incubation. Moreover, the strain HTA426 was able to grow and produce cellulase on alkali-treated sugarcane bagasse, rice straw and water hyacinth as carbon sources. Enzymatic hydrolysis of sugarcane bagasse, which was regarded as the most effective carbon source for cellulase production (CMCase activity = 103.67 U/mL), followed by rice straw (74.70 U/mL) and water hyacinth (51.10 U/mL). This strain producing an efficient thermostable cellulose is a potential candidate for developing a more efficient and cost-effective process for converting lignocellulosic biomass into biofuel and other industrial process.

## Introduction

The increasing of energy demands and environmental problems caused by the use of non-renewable fossil fuels, it has become necessary to introduce alternative energy sources. Bioethanol produced from lignocellulosic biomass is considered as the alternative and sustainable resource for renewable fuel, which can relieve the pressure of the energy crisis, and also helps to reduce greenhouse gas emissions [1–3]. Lignocellulosic biomass contains polymers of cellulose, hemicellulose and lignin bound together in a complex structure. These major components form a complex structure that is resistant to microbial and enzymatic activity. Due to the recalcitrant structure of plant cell walls, lignocellulosic raw materials must be pretreated before their enzymatic hydrolysis to monosaccharides [4, 5]. Various pretreatment methods such as alkaline [6], acid [7], and steam explosion [8] have been widely used. However, there still exist some problems in the pretreatment such as high temperature and pressure, resulting in low sugar yield [9, 10]. An alkaline pretreatment such as sodium hydroxide (NaOH) has been studied extensively to improve enzymatic hydrolysis [11]. The main advantage of the process is efficient removal of lignin from the biomass. Alkali pretreatment can be carried out at ambient conditions, but pretreatment times are on the order of hours or days rather than minutes or seconds. Compared with acid processes, alkaline processes cause less sugar degradation, and many of the caustic salts can be recovered and/or regenerated [12, 13]. Therefore, successful pretreatment of cellulosic material is an essential step for subsequent enzymatic hydrolysis of the substrate in order to obtain glucose that is converted to bioethanol by microorganisms.

Cellulases are a group of complex enzymes that catalyse the hydrolysis of cellulose to fermentable sugar. The complete enzymatic hydrolysis requires the synergistic action of endoglucanase (E.C. 3.2.1.4), exoglucanase (E.C. 3.2.1.91) and β-glucosidase (E.C. 3.2.1.21) to convert the cellulose into glucose monomers [14]. Recently, thermophilic bacteria have received considerable attention as source of cellulolytic enzymes. The hydrolysis of cellulose by the bacteria has several features such as: more stability, increased specific activity and facilitated mass transfer [15–17]. Therefore, thermophilic cellulose-degrading bacteria have been isolated from various environments such as soil [18–20], compost systems [21–23], and wastewaters [24].

The present study was to isolate a potential thermophilic cellulase-producing bacterium from a hot spring district. Subsequently, the cellulolytic enzyme was purified and characterized according to various parameters. Finally, the strain HTA426 was evaluated using lignocellulosic biomass (rice straw, sugarcane bagasse, and water hyacinth) as carbon sources for cellulase production.

## Materials and methods

### Ethics statement

No specific permits were required for the described field studies because no human or animal subjects were involved in this research. No specific permissions were required for these locations/activities. The sampling location was not privately owned or protected in any way, and the field studies did not involve endangered or protected species.

### Isolation and screening of thermophilic cellulase-producing bacteria

Soil samples were collected from hot spring district of Rucheng Reshui Town, Chenzhou, Hunan province, China (25°31'44.6"N and 113°54'52.8"E). The samples were taken from organic-rich soil. For bacteria isolation, 10 g of soil was added to 100 mL enrichment medium in 250 mL flask. The enrichment medium contained (g/L): peptone, 1.0; urea, 0.3; KH_2_PO_4_, 2; (NH_4_)_2_SO_4_, 1.0; CaCl_2_, 0.3; MgSO_4_.7H_2_O, 0.3; FeSO_4_.7H_2_O, 0.005; MnSO_4_.H_2_O, 0.016; ZnSO_4_.7H_2_O, 0.014 and CoCl_2_, 0.002; pH 7.0 [25]. The medium was supplemented with carboxymethyl cellulose (CMC) as a carbon source. The flask was kept in a shaking incubator at 60°C and 170 rpm for 24 h. After that, 1 mL of culture medium was transferred to fresh medium and the incubation was continued for another 24 h under the same conditions as above. This process was repeated for one week. Final cultures were used to isolate pure cellulose degrading bacteria by the serial dilution. Approximately, 0.1 mL of each dilution was spread onto a CMC agar plate containing enrichment medium and incubated at 60°C for 24 h. The single colony was re-streaked on plate to ensure purity. To screen for cellulase producing bacterial isolation, all the pure isolates were then spotted on CMC agar plates and incubated at 60°C for 24 h. The plates were stained with 1% (w/v) Congo-red solution for 15 min at room temperature and washed with 1 M NaCl for de-staining [26]. The qualitive measure of extracellulase activity is the presence of clear zone around the growing colony against the dark red background.

### Bacteria identification

The PCR amplification of the 16S rRNA gene was carried out using the universal primers 27F (5'-AGTTTGATCCTGGCTCAG-3') and 1492R (5'-GGTTACCTTGTTACGACTT-3'). The process of PCR was done under the following conditions: initial denaturation at 95°C for 4 min, denaturation at 94°C for 30 s, annealing at 55°C for 30 s, extension at 72°C for 90 s and final extension at 72°C for 7 min and then 4°C forever. PCR amplified products were then purified and sequenced. The similarity search for the sequence was carried out using the BLAST program of the National Center of Biotechnology Information (NCBI). Phylogenetic tree was constructed by applying the neighbor-joining method using CLUSTAL software in MEGA programme [27]. In order to estimate the confidence of the tree topologies, bootstrap resampling analysis for 1000 replicates was performed.

### Effect of fermentation medium on cellulase production

To study the effect of broth mediums on cellulase enzyme production, the following three mediums, Mandel and Reese medium [25], Minimum Salt medium [28] and Bushnell Haans medium [29] were investigated. The isolated bacteria strain was first grown in 100 mL of enrichment medium in 250 mL flask and incubated at 60°C under shaking condition at 170 rpm until the exponential grown phase was reached. The cultures were transferred into 100 mL of different mediums in 250 mL flask and incubated for 72 h under the same condition as above. The supernatant was collected for CMCase activity assay.

### Effect of incubation period on cellulase production

After selection of broth mediums, the effect of incubation periods on cellulase enzyme production was also investigated. The isolated strain was grown in enrichment medium as described above until exponential phase. Then, the cultures were transferred into 100 mL of Mandel and Reese medium in 250 mL flask and incubated at 60°C under shaking condition at 170 rpm. The supernatant was collected at 24 h intervals up to 168 h. The CMCase activity was estimated using DNS reagent as discussed in the enzyme assay section.

### Purification of cellulase enzyme

All the steps of purification were performed at 4°C. After the cultivation, bacterial culture was centrifuged at 10,000 rpm for 20 min to remove the cells and residual medium. The enzyme was precipitated with ammonium sulfate (80% saturation) for overnight, and recovered by centrifugation at 10,000 rpm for 20 min. The pellet was resuspended in a small amount 100 mM phosphate buffer, pH 7.0 and dialyzed overnight against the sample buffer. The sample was then concentrated using centricon tube (10 kDa cut-off) and applied to anion exchange chromatography using DEAE-cellulose column (1.6×30 cm) previously equilibrated with 5 volumes of 100 mM phosphate buffer, pH 7.0. The column was eluted with a linear gradient of 0–0.5 M NaCl in the same buffer at a flow rate of 0.5 mL/min. Protein peaks were detected by measuring the optical density at 280 nm using UV-VIS spectropmetehotor. Fractions with cellulase activity were collected and subjected to enzyme activity assay and protein content. The specific activity was calculated and the values were expressed in the terms of fold of purification. The fraction with the maximum specific activity was selected and stored at 4°C for further analysis.

### Polyacrylamide gel electrophoresis

Sodium dodecylsulfate polyacrylamide gel electrophoresis (SDS–PAGE) was performed with 12.5% resolving and 4% stacking gels according to the method of Laemmli [30]. After electrophoresis, the gel was stained with Coomassie Brilliant blue. For zymogram analysis, 0.1% (w/v) CMC was incorporated into the polyacrylamide during gel preparation. The electrophoresis was performed at 4°C. The gel was then soaked in 100 mM phosphate buffer, pH 7.0 for 1 h prior to staining with 0.2% (w/v) Congo red. The gel was destained with 2 M NaCl until the cellulase activity was visualized as clear band against the red background [31].

### Enzymatic assay

CMCase activity was determined by measuring the amount of reducing sugar liberated from CMC using 3,5-dinitrosalicylic acid (DNS) method [32]. The reaction mixture was prepared by mixing 50 μL of crude enzyme solution with 50 μL of 1% (w/v) CMC dissolved in 100 mM phosphate buffer, pH 7.0. The mixtures were incubated at 50°C for 20 min. The reactions were stopped by adding 0.3 mL of DNS reagent. All the mixtures were heated in boiling water at 100°C for 5 min for color development. Subsequently, 0.6 mL of distilled water was added to stop the reaction, cooled at room temperature and the optical density was measured at 575 nm. All of CMCase assay were performed in three replicated. The enzyme activity was determined by using a calibration curve for glucose. One unit (U) of the enzyme activity was defined as the amount of enzyme that released 1 μmol of glucose per minute. Protein concentration was determined by Bradford method [33], using bovine serum albumin (BSA) as a standard for the calibration curve.

### The optimum pH and pH stability

The optimum pH of the purified cellulase was determined by incubating the mixture of the purified enzyme and 1% (w/v) CMC in the presence of appropriate buffers; 100 mM acetate buffer (pH 3.0–6.0), 100 mM phosphate buffer (pH 7.0–8.0) and 100 mM Tris-HCl buffer (pH 9.0–10.0). The reaction mixtures were incubated at 50°C for 60 min. The pH stability was determined by incubating the purified enzyme and respective buffers having different pH ranging from 3.0 to 10.0 at 50°C for a period of 5 h. The residual activity of each sample for hydrolysis of CMC was then estimated under assay conditions as described above. For the determination of pH stability, the purified enzyme was incubated in buffers having different pH ranging from pH 7.0 to 12.0 for 1 h at 40°C

### Effect of temperature and stability

The optimum temperature of the purified enzyme was determined by incubating the mixture of the purified enzyme and 1% (w/v) CMC in 100 mM phosphate buffer, pH 7.0 for 60 min at different temperatures ranging from 30°C to 90°C. The heat stability of the purified enzyme was determined by incubating the purified enzyme in 100 mM phosphate buffer, pH 7.0 for 60 min at temperatures ranging from 30°C to 90°C for a period of 5 h. The residual activity of each sample for hydrolysis of CMC was then estimated under assay conditions as described above.

### Effect of various additives on enzyme activity

The effect of various additives on the purified enzyme was determined by the presence of metal ions and surfactants. The metal ions used in this study were NaCl, KCl, CaCl_2_, ZnSO_4_ and CuSO_4_, and surfactants including sodium dodecyl sulphate (SDS), Triton X-100 and Tween 80. The concentration of each additive was 5 mM. The reaction mixtures with various additives were incubated for 60 min at 60°C and the CMCase activity was estimated under assay conditions as described above.

### Lignocellulosic biomass pretreatment and chemical composition analysis

Lignocellulosic biomass used in this study were rice straw, sugarcane bagasse, and water hyacinth. The samples were dried, ground to powder, and stored in a sealed plastic bag at room temperature. To prepare the lignocellulosic materials hydrolysis, all of the samples were chemically pretreated using 3% (w/v) NaOH solution (solid to liquid ratio of 1:20) at 50°C for 2 h. The pretreated samples were then washed with distilled water to remove the unwanted chemicals until the wash water becomes neutral. The samples were then filtered by suction filtration with Buchner funnel, dried, and stored for further use. The compositional determination of lignocellulosic biomass (cellulose, lignin, and holocellulose) were determined using the 20% nitric acid–ethanol, 72% sulfuric acid, and sodium chlorite methods, respectively [34].

### Enzymatic hydrolysis

To investigate the effect of biomass concentrations on cellulase production, isolate strain was grown in Mandel and Reese medium supplemented with pretreated rice straw, sugarcane bagasse and water hyacinth as a carbon source. The isolate strain was first carried out in 100 mL of in medium containing one of the above carbon sources. After the exponential phase of growth, the cultures were transferred in a fresh medium supplemented with 1%, 3% and 5% (w/v) of the same carbon source, and incubated at 60°C under shaking condition at 170 rpm. The CMCase activity was investigated at 24 h intervals up to 216 h by using DNS reagent as discussed in the enzyme assay section.

## Results and discussion

### Isolation, screening and identification of thermophilic cellulase-producing bacteria

**Fig 1:**
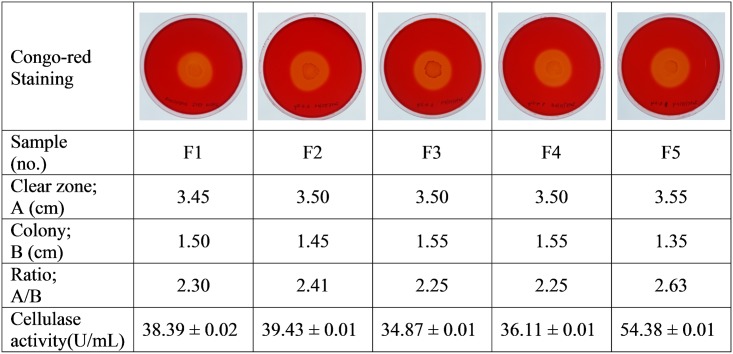
Screening for cellulolytic activity of five bacterial strains on CMC agar plate and fermentation liquid medium.

**Fig 2:**
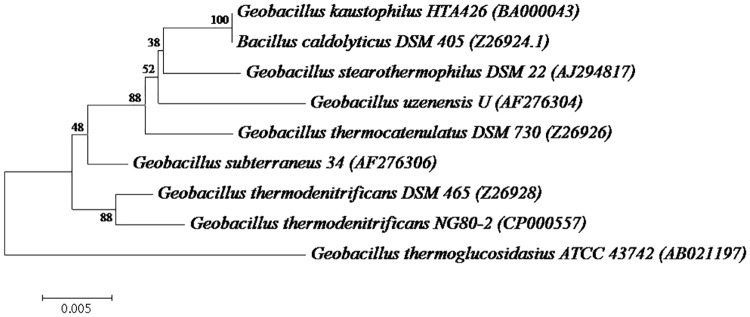
Phylogenetic tree of *Geobacillus* sp. HTA426 associated with other members of the genus *Geobacillus* sp. and *Bacillus* sp.; The tree was obtained using the 16S rRNA sequence retrieved from the database by using the neighbor-joining method. The bootstrap values were generated from 1000 replicates.

Microorganisms hydrolyzing CMC were isolated and screened for their cellulolytic potential on CMC agar plates. Five bacterial strains exhibited cellulolytic activity, as revealed by the formation of a clear zone on the screening medium. Base on the calculation of the ratio of the diameter of the zone of clearance to the diameter of the colony, it was determined that these bacterial isolates demonstrated differences in their ability to degrade CMC (Fig 1). However, the diameter of the hydrolyzing zone may not accurately reflect the real cellulase activity [35]. Thus, the isolates were screened according to the cellulase activity level in liquid fermentation medium. Based on its maximum zone of clearance (F5), isolate HTA426 exhibited higher CMCase activity (54.38 ± 0.01 U/mL) than the other strains did, and it was selected for further identification and characterization. The phylogenetic analysis, which was based on a BLAST search and bootstrapping of 1000 replicates of the 16S rRNA gene sequence, revealed that the HTA426 strain formed a clade with *Bacillus caldolyticus* DSM 405 (Fig 2). The degree of sequence similarity of strain HTA426 to *Bacillus* sp. DSM 405 was 99%. In previous studies, *Geobacillus* sp. have been reported for producing the cellulase enzyme, including *Geobacillus* sp. WSUCE1 [17], *Geobacillus* sp. T1 [20], and *Geobacillus* sp. T4 [24]. However, HTA426 strain has not been yet reported to produce cellulase enzyme, therefore, it is a novel cellulase producing bacterial strain.

### Effect of fermentation media and incubation time on cellulase production

**Fig 3:**
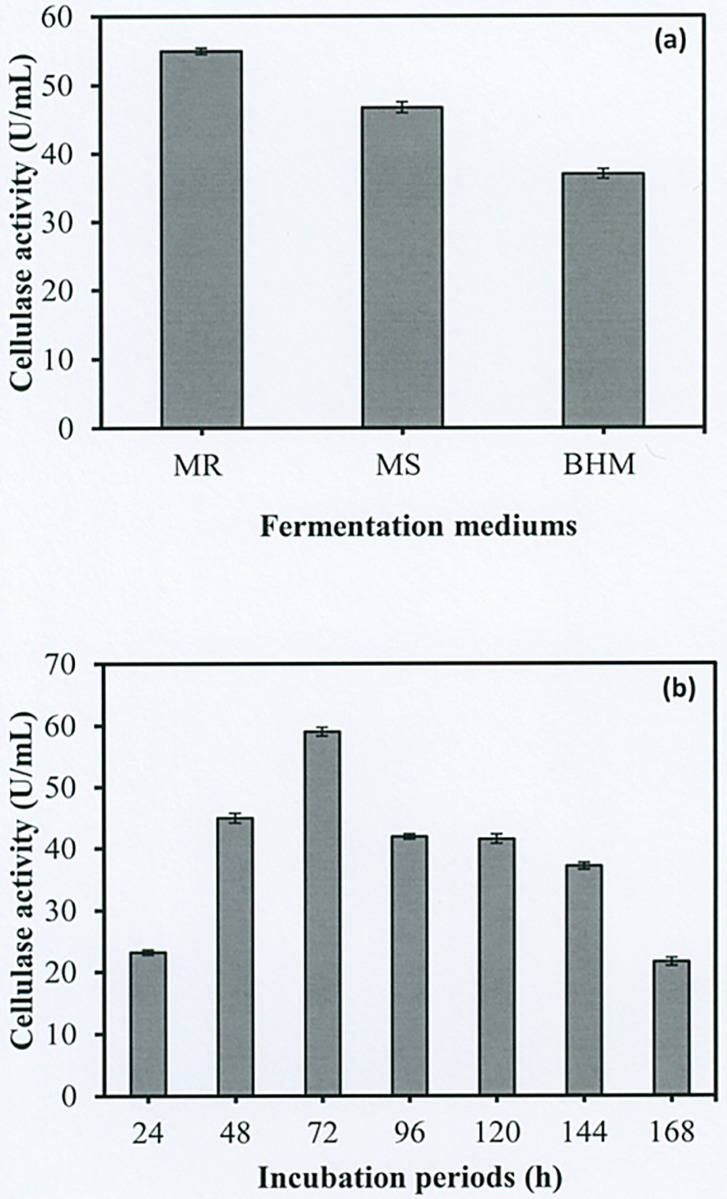
Effect of culture media and incubation periods on cellulase production by *Geobacillus* sp. HTA426. (a) Isolated strain cultured in Mandel and Reese medium (MR), Minimum Salt medium (MS), and Bushnell Haas medium (BHM) supplemented with CMC as the sole carbon source. (b) Isolated strain cultured in Mandel and Reese medium at 60°C. CMCase activity was investigated at 24 h intervals for up to 168 h by using DNS reagent.

To select the optimal fermentation medium, cellulase production was estimated by growing the bacterial isolate in different culture media. The results revealed that Mandel and Reese medium (CMCase activity = 54.99 U/mL) was optimal for maximizing the production of cellulase by using *Geobacillus* sp. HTA426, followed by Minimum Salt medium (CMCase activity = 46.70 U/mL) and Bushnell Haas medium (CMCase activity = 36.94 U/mL), respectively (Fig 3a). However, Minimum Salt medium and Bushnell Haas medium contained with nitrate, which may interfere with the cellulase production. Rajmane and Koreler (2012) reported that nitrites and nitrates containing media are toxic for cellulase production [36]. Moreover, Chaurasia et al. (2013d) having also similar opinion as they have reported that nitrate containing media are very toxic to several members of microorganisms [37]. Hence, in the present study, the Mandel and Reese medium was selected to investigate the incubation periods. A time course of the bacterial strain and enzyme production was performed over a period of 168 h. The results revealed that the HTA426 strain can produce cellulase within a short incubation period. As shown in Fig 3b, 72 h of incubation was the optimal incubation period for maximizing the production of cellulase. However, increasing the duration of the incubation to 168 h had no effect on cellulase production, as demonstrated by the gradual reduction in enzyme activity. This phenomenon might be attributable to the depletion of nutrients in the medium, which stresses bacterial physiology, resulting in the inactivation of the secretory machinery of the enzymes [38].

### Purification of cellulase enzyme

**Fig 4:**
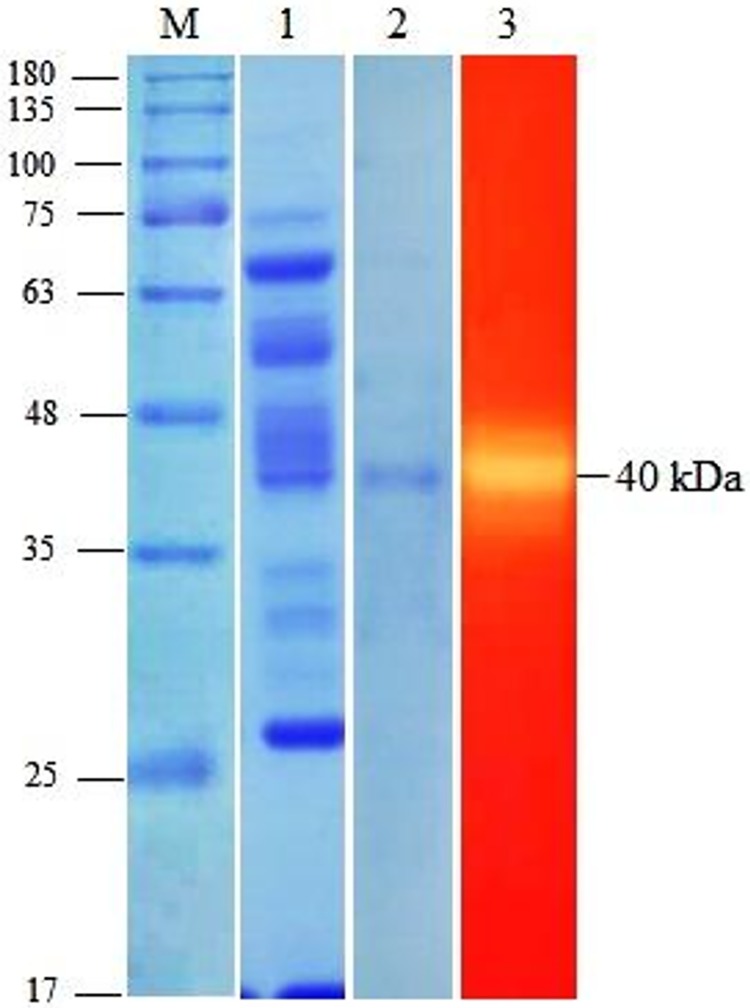
SDS-PAGE and zymogram of the purified cellulase produced by *Geobacillus* sp. HTA426. M: protein maskers, Lane 1: ammonium sulfate precipitate of culture supernatant, Lane 2: fraction with highest cellulase activity collected from ion exchange chromatography, Lane 3: zymogram of the cellulase activity of the purified enzyme. Based on the gels, the molecular weight of the enzyme was estimated to be 40 kDa.

The crude enzyme extract was first partially purified using 80% ammonium sulfate precipitation. Data showed that the ammonium sulfate fractionation led to a total protein content of 1013.03 mg and total activity of 3009.54 U, resulting in specific activity of 2.97 U/mg (Table 1). After DEAE-cellulose ion exchange chromatography, the specific activity was increased to 6.76 U/mg, resulting in approximately 5.12-fold purification with a recovery yield of 10.14%. The molecular weight of the purified cellulase was estimated to be about 40 kDa as confirmed by the presence of the single protein band in native zymogram. The result of activity staining has also shown the active band of cellulase enzyme corresponding to the size of about 40 kDa (Fig 4).

**Table 1:** Summary of purification of the cellulase produced by *Geobacillus* sp. HTA426.

Purificationsteps	Total activity (U)	Total protein (mg)	Specific activity (U/mg)	Purification (fold)	Yield (%)
Crude	6796.94	5145.74	1.32	1.00	100
Ammonium sulfate	3009.54	1013.03	2.97	2.25	44.28
DEAE-cellulose	689.116	101.91	6.76	5.12	10.14

### Effect of pH on enzyme activity and stability

**Fig 5:**
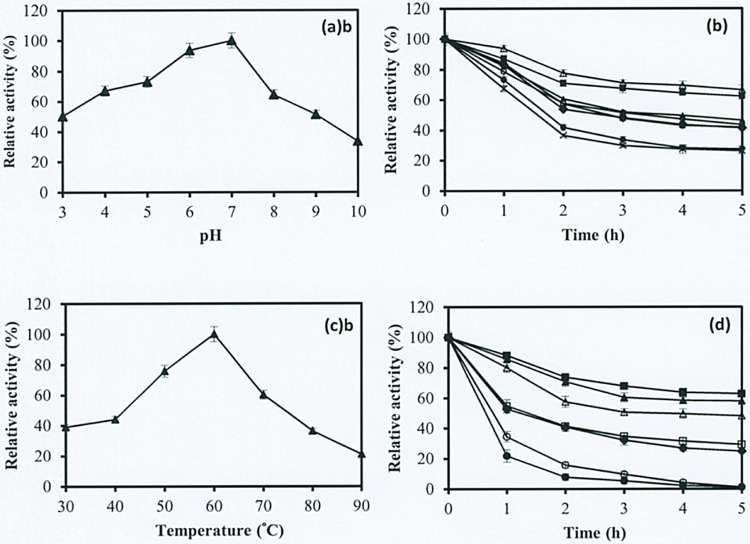
Effect of (a) pH on enzyme activity, (b) pH on enzyme stability; pH 3.0 (♦), pH 4.0 (□), pH 5.0 (▲), pH 6.0 (■), pH 7.0 (Δ), pH 8.0 (○), pH 9.0 (●), and pH 10.0 (×), (c) temperature on enzyme activity, and (d) temperature on enzyme stability; 30°C (♦), 40°C (□), 50°C (▲), 60°C (■), 70°C (Δ), 80°C (○), and 90°C (●).

The effect of pH on the CMCase activity of the purified cellulase was determined at pH values ranging from 3.0 to 10.0. The activity profile of the purified cellulase demonstrated the highest enzyme activity at pH 7.0 in phosphate buffer, and the enzyme was still active over a wide pH range. More than 50% of the maximum CMCase activity of the purified cellulase was observed from pH 3.0 to 9.0, whereas the minimum CMCase activity was observed at alkaline pH 10.0, with only 34% (Fig 5a). However, for *Geobacillus* sp. T1 and *Geobacillus* sp. T4, previous studies have observed the maximum CMCase activity at pH 6.5 and 7.0 [20, 24].

For the pH stability of the purified cellulase was determined at different pH values, as indicated earlier in the text. As shown in Fig 5b, more than 40% of the maximum CMCase activity was retained at pH values ranging from 3.0 to 8.0 after 5 h of incubation. This result indicated the pH stability of cellulase produced by *Geobacillus* sp. HTA426 over a wide pH range, and that the pH did not influence enzyme stability after prolonged incubation.

### Effect of temperature on enzyme activity and stability

The effect of temperature on CMCase activity was also determined at different temperatures ranging from 30°C to 90°C, as shown in Fig 5c. The maximum CMCase activity of the purified cellulase was obtained at 60°C. CMCase activity increased linearly with the temperature up to 60°C but gradually declined above this temperature. At 80°C and 90°C, CMCase activity declined rapidly, possibly because of thermal denaturation.

However, in industrial processes where enzymes are used, the problem of thermal inactivation of the enzyme is often encountered [39]. Hence, enzyme stability is a crucial factor for industrial applications. In the current study, the thermal stability of the purified cellulase was determined at temperatures ranging from 30°C to 90°C, as shown in Fig 5d. More than 80% of the maximum CMCase activity of the purified enzyme was maintained at temperatures of 50°C to 70°C after 1 h of preincubation at pH 7.0, and only approximately 50% of the maximum CMCase activity was lost after 5 h of incubation. However, more than 40% of the CMCase activity of cellulase was stable at a wide temperature range of 30°C to 70°C after 2 h of incubation. However, at 80°C and 90°C, the CMCase activity of the enzyme rapidly decreased after 5 h of incubation because of the increase in the incubation time; enzyme denaturation increased at higher temperatures. Tai et al. (2004) reported that the CMCase activity of *Geobacillus* sp. dropped immediately even with a slight increase of temperature above 70°C, suggesting that the enzyme was highly sensitive to temperatures higher than 70°C [24]. After 1 h incubation, the CMCase activity of cellulase produced by *Geobacillus* sp. retained 90% and 60% of the maximum activity at 70°C and 80°C, respectively. However, several other thermophilic strains have been shown to produce thermostable cellulase, but their produced cellulase does not retain activity at higher temperatures (e.g., ≥60°C) for prolonged periods [40].

### Effect of various additives on enzyme activity

The effect of metal ions and surfactants on enzyme activity were determined by incubating the individual ions or surfactants with enzyme reaction. The CMCase activity of cellulase strongly increased in the presence of CaCl_2_ (174.32%) followed by NaCl (158.11) and KCl (117.57%), respectively, whereas the CMCase activity was slightly inhibited by ZnSO_4_ (97.29%) and CuSO_4_ (85.14%). Gaur and Tiwari (2015) had also reported that CMCase activity was strongly stimulated by CaCl_2_, NaCl and KCl ions [41]. However, the inhibition of cellulase by CuSO_4_ and ZnSO_4_ ions could bind the thiol groups and interact with imidazole or carboxyl groups of amino acids, resulting in the decreased of enzyme activity [42].

**Fig 6:**
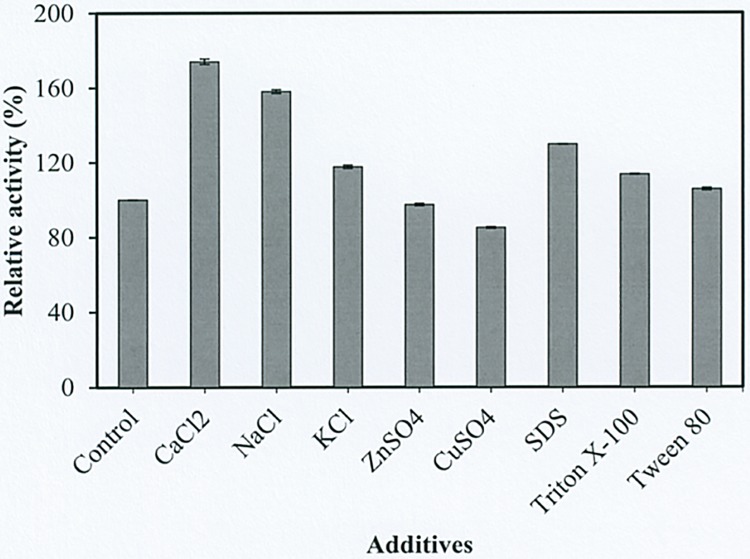
Effect of various additive on enzyme activity.

The anionic and non-ionic surfactants were found as activators of the cellulase enzyme as the CMCase activity increased with SDS (129.73%), Triton X-100 (113.51%) and Tween-80 (105.41%), respectively (Fig 6). The increase in the CMCase activity with these surfactants has also been observed by Seki et al. (2015) and Asha et al. (2012) [43, 44]. It might be due to the surfactants have the capability to modify the surface property and help to minimize the irreversible inactivation of cellulase. Therefore, these surfactants have been found to stimulate the cellular enzyme [45].

### Effect of alkaline pretreatment on biomass composition

The biomass pretreatment is aimed to fractionate the hemicellulose and lignin contents to reduce the cellulose crystallinity and increase the porosity of the materials for subsequent depolymerization process [46, 47]. In the present study, the alkaline pretreatment was efficient in reducing the hemicellulose and lignin contents (sugarcane bagasse: 28.83% to 9.85% and 18.96% to 4.25%; rice straw: 32.16% to 12.10% and 16.32% to 3.55%; water hyacinth: 33.63% to 37.90% and 26.13% to 7.40%, respectively. Simultaneously, there was an increased in the cellulose content (sugarcane bagasse: 35.83% to 76.80%; rice straw: 32.36% to 75.45%; water hyacinth: 19.63% to 40.15%, respectively. The solids recovery for all pretreated biomass were more than 80% (Table 2). These results are in agreement with those reported by Lima et al. (2014), who have been used acid, alkaline, sulfite and hot water pretreatment in sugarcane bagasse and several grasses including *P*. *purpureum* [48]. The results demonstrated that acid pretreatment was the most effective compound for hemicellulose removal, whereas alkaline pretreatment was more suitable for removing hemicellulose and lignin content in *P*. *purpureum*.

**Table 2:** Chemical composition of untreated and pretreated lignocellulosic biomass.

Biomass	Compositions (%)				
		Cellulose	Hemicellulose	Lignin	Solids recovered (%)
Sugarcane	Untreated	35.83 ± 1.79	28.83 ±1.22	18.94 ± 0.32	100.00
	Pretreated	76.80 ± 0.57	9.85 ± 0.49	4.25 ± 0.28	82.64
Rrice straw	Untreated	32.36 ± 0.59	32.16 ± 0.32	16.32 ± 0.32	100.00
	Pretreated	75.45 ± 0.52	12.10 ± 0.69	3.55 ± 0.21	84.17
Water hyacinth	Untreated	19.63±1.80	33.63 ± 0.81	26.13 ± 0.19	100.00
	Pretreated	40.15±0.21	37.90 ± 0.23	7.40 ± 0.46	81.28

### Effects of solid loading on the cellulase enzyme production

**Fig 7:**
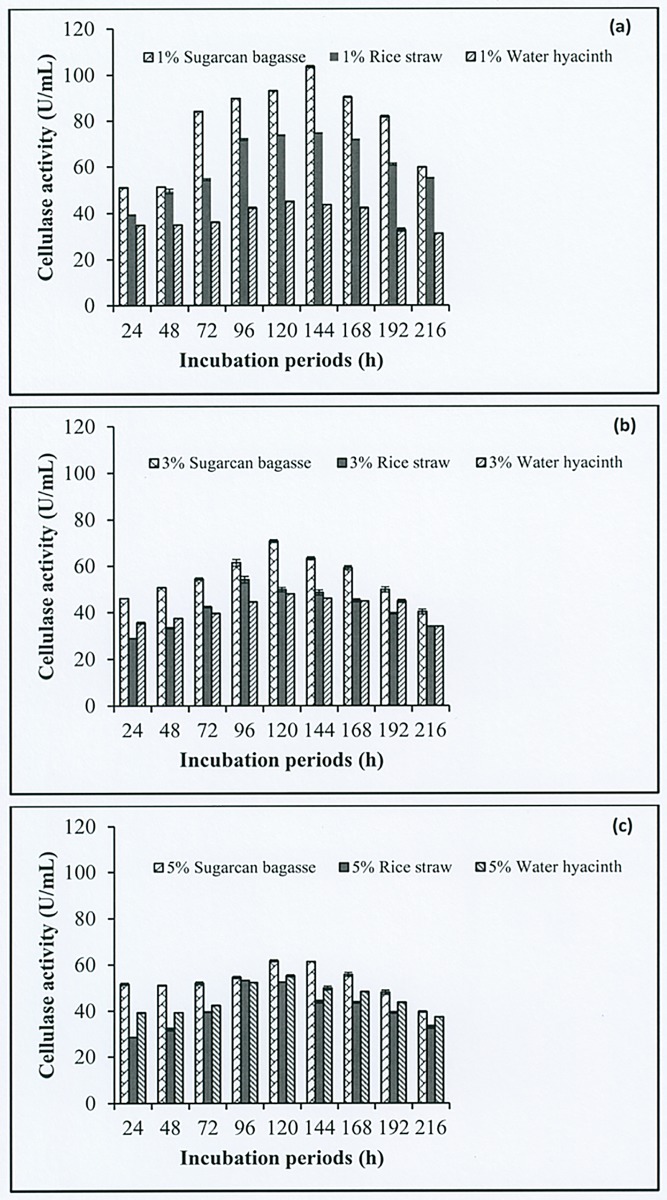
Effect of incubation periods and concentrations; (a) 1%, (b) 3% and (c) 5% of pretreated sugarcane bagasse, rice straw, and water hyacinth on cellulase production by *Geobacillus* sp. HTA426.

The effect of different solid loading on the cellulase production by *Geobacillus* sp. HTA426 was investigated using pretreated sugarcane bagasse, rice straw, and water hyacinth. Of the various solid loading concentrations (1%, 3%, and 5% w/v) used in the medium, the presence of a 1% solid loading (Fig 7a) resulted in the highest CMCase activity of cellulase production for sugarcane bagasse (103.67 U/mL), followed by rice straw (74.70 U/mL); the highest CMCase activity for water hyacinth (55.10 U/mL) was observed in the presence of a 5% solid loading (Fig 7c). The CMCase activity of cellulase production in the presence of a 3% solid loading of sugarcane bagasse, rice straw, and water hyacinth is shown in Fig 7b. The lowest CMCase activity for sugarcane bagasse and rice straw was observed in the presence of a 5% solid loading (Fig 7c), that for water hyacinth was observed in the presence of a 1% solid loading. However, the decrease in CMCase activity beyond the maximum solid loading concentration, that is 5% (sugarcane bagasse and rice straw), may be due to the presence of inhibitors. This is supported by the findings of Oguntimein and Moo-Young (1991), who reported the inhibitory effect of accumulated cellobiose and cellodextrin with a low degree of polymerization [49]. Moreover, the high solid loading also increases the viscosity of the slurry, and can result in poor mixing and mass transfer problem that reduce sugar conversion [50, 51].

To select the optimal duration for cellulase production by *Geobacillus* sp. HTA426, bacterial isolate was grown in the solid loading of sugarcane bagasse, rice straw, and water hyacinth for up to 216 h. Over a period of up to 144 h, the highest CMCase activity was observed in sugarcane bagasse (103.67 U/mL) and rice straw (74.70 U/mL) (Fig 7a). Up to 120 h, the highest CMCase activity was observed in water hyacinth (55.10 U/mL) (Fig 7c). Therefore, the lignocellulosic biomass resources are a potential carbon source for cellulase production as well as for conversion to biofuels.

## Conclusion

The thermophilic bacterial strain HTA426, which exhibits cellulolytic potential, was isolated from a sample taken from a hot spring district and was identified as *Geobacillus* sp. The cellulase enzyme produced by the *Geobacillus* sp. HTA426 was purified through ammonium sulfate precipitation and ion exchange chromatography. The molecular weight of the purified enzyme was determined to be 40 kDa. This enzyme was stable over a wide range of pH and temperature, which is another valuable characteristic of industrial enzymes. Moreover, the HTA426 strain can produce cellulase when grown on various lignocellulosic biomass sources such as pretreatd rice straw, sugarcane bagasse, and water hyacinth. Therefore, *Geobacillus* sp. HTA426 can be used for cost-efficient cellulase production for bioconversion of lignocellulosic biomass into biofuels.
